# From Green Technology to Functional Olive Oils: Assessing the Best Combination of Olive Tree-Related Extracts with Complementary Bioactivities

**DOI:** 10.3390/antiox10020202

**Published:** 2021-01-30

**Authors:** Álvaro Hernáez, Sara Jaramillo, Aránzazu García-Borrego, Juan Antonio Espejo-Calvo, María-Isabel Covas, Gemma Blanchart, Rafael de la Torre, Alegría Carrasco-Pancorbo, María Dolores Mesa, Maria África Fernández-Prior, Olga Castañer, Montserrat Fitó

**Affiliations:** 1 Cardiovascular Risk and Nutrition Research Group, Hospital del Mar Medical Research Institute (IMIM), Carrer Doctor Aiguader 88, 08003 Barcelona, Spain; hernaez@clinic.cat (Á.H.); gblanchart@imim.es (G.B.); 2 Cardiovascular Risk, Nutrition, and Aging Research Group, August Pi i Sunyer Biomedical Research Institute (IDIBAPS), Carrer Mallorca 183, 08036 Barcelona, Spain; 3 Consorcio CIBER, M.P. Fisiopatología de la Obesidad y Nutrición (CIBERObn), Instituto de Salud Carlos III, Calle Monforte de Lemos 3-5, Pabellón 11, 28029 Madrid, Spain; 4 Department of Food Phytochemistry, Instituto de la Grasa (Consejo Superior de Investigaciones Científicas—CSIC), Campus Universitario Pablo de Olavide—Edificio 46, Ctra. de Utrera, km. 1, 41013 Seville, Spain; smjaramillo@ig.csic.es (S.J.); aranzazu@ig.csic.es (A.G.-B.); 5 Vegetable By-Products of Mediterráneo S.L., Cl Isla Menor CEP Jose Maria Blanco SN, 41010 Seville, Spain; 6 Instituto para la Calidad y Seguridad Alimentaria S.L. (ICSA)-TECNOFOOD I+D Soluciones S.L., Avda. de la Hispanidad, 17, 18320 Granada, Spain; jaespejo@hotmail.com; 7 NUPROAS Handelsbolag, Apartado de Correos 93, 17242 Girona, Spain; maria.nuproas@gmail.com; 8 Integrative Pharmacology and Systems Neuroscience Research Group, Hospital del Mar Medical Research Institute (IMIM), Carrer Doctor Aiguader 88, 08003 Barcelona, Spain; rtorre@imim.es; 9 Department of Experimental and Health Sciences, Universitat Pompeu Fabra (CEXS-UPF), Carrer Doctor Aiguader 88, 08003 Barcelona, Spain; 10 Department of Analytical Chemistry, Faculty of Science, University of Granada, Avenida Fuentenueva s/n, 18071 Granada, Spain; alegriac@ugr.es; 11 Department of Biochemistry and Molecular Biology II, Institute of Nutrition and Food Technology “José Mataix”, Centro de Investigación Biomédica, University of Granada, Avenida de la Investigación s/n, 18016 Granada, Spain; mdmesa@ugr.es; 12 Instituto de Investigación Biosanitaria de Granada, Avenida Fuerzas Armadas 2, 18014 Granada, Spain

**Keywords:** olive tree, phenolic compound, triterpenes, bioactivity, functional olive oil, 3,4-dihydroxyphenylglycol, hydroxytyrosol, oleuropein, oleocanthal

## Abstract

Our aim was to assess the combination of olive tree-related extracts with the most favorable profile of in vitro bioactive properties. We tested the antioxidant (increment of low-density lipoprotein resistance against oxidation), vasoactive (promotion of nitric oxide release and decrease of endothelin-1 production in human umbilical vein endothelial cells), anti-inflammatory (decrease of the endothelial production of vascular cell adhesion molecule-1), and antithrombotic (reduction of the endothelial release of plasminogen activator inhibitor-1) capacities of six phenolic extracts and three triterpenic acid solutions (Ps and Ts, respectively). We tested extracts alone and in combination, at nutritional (Ps: 0.05–0.5 μmol/L; Ts: 0.001–0.1 μmol/L) and nutraceutical doses (Ps: 1–10 μmol/L; Ts: 0.25–10 μmol/L). The combination of Ps rich in 3,4-dihydroxyphenylglycol (76%, P2), hydroxytyrosol (95%, P3), and oleuropein (70%, P4) (final nutritional concentration: 0.15 μmol/L; final nutraceutical concentration: 3 μmol/L) was the best in order to prepare functional products and nutraceuticals with cardioprotective properties, despite the fact that the isolated extract with the greatest in vitro properties was P5 (75% oleocanthal), suggesting a potential synergistic effect among different olive components.

## 1. Introduction

Olive mill waste such as olive pomace (a mixture of minerals, fatty acids, sugars, and phenolic compounds) contains high quantities of organic acids (e.g., phenolic acids) that have been intensively studied because of their phytotoxic potential [1,2,3]. However, they are also a source of valuable nutritional resources [1,4]. Valorization of these residual materials as functional ingredients is a required innovation step and a challenge for the food industry. These by-products can be used as a source of healthy components and, in parallel, for decreasing the environmental impact of residues of olive oil production [1]. The by-products must, however, be characterized, and an assessment of their bioactivities is required [5].

Whereas approximately 2% of olive phenolic compounds are transferred to oil in the production process, 98% of them are retained in the cake [6] which is an ideal source of bioactive compounds for the preparation of enriched functional foods. Olive phenolic compounds are essential biological compounds in virgin olive oil [7] and have been extensively investigated due to their biological effects on oxidative stress and low-grade inflammation [8], endothelial dysfunction [9,10], immune cell gene expression [9], and thrombosis responses [9]. They have also been used for the enrichment of functional olive oils with additional health properties [11]. Other olive-derived compounds associated with health effects include terpenoids such as oleanolic and maslinic acids and biosynthesized intermediate products [12]. Most of the biological effects of these compounds have been tested either collectively [8] or individually [13,14,15]. However, to the best of our knowledge, no study has described to date an evidence-based strategy to develop a functional food with optimal pre-clinical characteristics. In addition, whether the individual effects of olive mill waste-derived products could act additively, synergistically, or antagonistically when combined remains to be established.

Our aim was to assess which combination of olive oil minor components (phenolic compounds and triterpenes) presented the most favorable profile in terms of in vitro bioactive properties (antioxidant, anti-inflammatory, anti-coagulant, and vasoactive capacities). Combinations were examined at doses related to real-life ones (in order to design a functional olive oil) or higher for the production of nutraceuticals.

## 2. Materials and Methods

### 2.1. Extracts of Bioactive Components of the Olive Tree

#### 2.1.1. Phenolic Compound Extracts

We elaborated six extracts of phenolic compounds from natural olive oil matrices (coming from olive oil solid waste or *alperujo*—produced in the Almazara San Francisco de Asís S. Coop. And., Montefrío, Granada, Spain—and olive tree leaves –picual variety from Montefrío, Granada, Spain). Phenolic extract #1 (P1) contained oleuropein (28%), 3,4-didroxyphenylglycol (20%), rutin (13%), luteolin-7-glucoside (8%), hydroxytyrosol (6.5%), and verbascoside (4%). It was obtained by a combination of chromatographic extractions, system that is in the process of being patented. Phenolic extract #2 (P2) contained 3,4-dihydroxyphenylglycol (76%), hydroxytyrosol (10%), luteolin-7-glucoside (5%), chlorogenic acid (3%), and vanillin (2%). It was obtained by the method described in the patent PCT/ES2012/070491 [16]. Phenolic extract #3 (P3) contained hydroxytyrosol (95%) and tyrosol (5%), and was obtained by the patented system PCT/ES2002/00058 [17,18] in which ionic resins in combination with membranes were used. Finally, phenolic extract #4 (P4) contained oleuropein (70%), vanillin (7.5%), luteolin-7-glucoside (5%), ferulic acid (4.5%), and verbascoside (2%). It was obtained from the P2 after a chromatographic fractionation by an AmberChrom™ CG161M resin in a system in the process of being patented. The phenolic composition of the different extracts was analyzed by high performance liquid chromatography coupled with a diode array UV detector and mass spectrometry (HPLC-DAD-MS). Samples were separated by a Jasco-LC-Net II ADC liquid chromatograph system using a MEDITERRANEA SEA C-18 reverse-phase analytical column (Teknokroma, Spain). We used water with 1% formic acid and acetonitrile with 1% formic acid as mobile phases (flow rate: 1 mL/min), increasing the proportion of the acetonitrile solution from 30% to 100% for the first 20 min, maintained at 100% for 10 min, and returned to 30% in 5 min. Spectra were recorded in the 200–600 nm range and the chromatograms were acquired at 280 nm (for simple phenolic compounds), 360 nm (for flavonoid glycosides), and 370 nm (for flavonoid aglycones), using pure compounds as reference materials for calibration.

Two additional extracts (#5 and #6) were obtained from virgin olive oil and from olive oil waste water produced in the Almazara San Francisco de Asís S. Coop. And. (Montefrío, Granada, Spain). The extract containing oleocanthal as the main compound was isolated from a phenolic concentrate prepared by liquid/liquid extraction from virgin olive [19]. The concentrate was separated by two consecutive chromatographic procedures [20]. First, we performed a separation of the phenolic extract in a silica gel column eluted with mixtures of dichloromethane and methanol (increasing methanol content up to 10%). Fractions containing oleocanthal were pooled and dried under vacuum, and subsequently purified in a Shimadzu HPLC SPD-10A system with a Kinetex C-18 column (5 µm, 100 A, 250 × 21.2 mm; Teknokroma, Spain) coupled with a DAD. Chromatographic separation was performed by elution gradient using water and increasing concentrations of methanol. Fractions were pooled and dried under vacuum and the residue was dissolved in dimethyl sulfoxide in order to obtain phenolic extract #5 (P5), containing oleocanthal (75%) and one unidentified compound (25%). Hydroxytyrosol was extracted by the procedure described by Fernandez-Bolaños et al. [16]. The isolated fractions were dried and then dissolved in dimethyl sulfoxide to obtain phenolic extract #6 (P6) which contained hydroxytyrosol (73%) and two unidentified compounds (27%). We confirmed the structure of the isolated compounds and their purity as described by García et al. [21], in a HPLC/electrospray ionization/MS system. It consisted of a Dionex Ultimate 3000RS U-HPLC (Thermo Fisher Scientific, Waltham, MA, USA), a MEDITERRANEA SEA C-18 column (Teknokroma, Sant Cugat del Vallès, Spain), and a microTOF-QII High Resolution Time-of-Flight mass spectrometer with qQ Time-of-Flight geometry (Bruker Daltonics, Bremen, Germany). We confirmed the structure of the compounds by tandem MS/MS spectra with HyStarTM 3.2. software (Bruker Daltonics, Bremen, Germany) [16,19,20].

#### 2.1.2. Triterpenic Acid Solutions

We purchased commercial lyophilizates of oleanolic, maslinic, and ursolic acids (references 42515, 68594, and 89797, respectively; Sigma-Aldrich, Barcelona, Spain), and suspended them in dimethyl sulfoxide to obtain three solutions of triterpenic acids: one with oleanolic acid (100%) (T1), one with maslinic acid (100%) (T2), and one with ursolic acid (100%) (T3).

#### 2.1.3. Tested Concentrations

Regarding phenolic compounds, we used as a physiological reference the maximal concentration of hydroxytyrosol measured in plasma after a standard dietary intake of a virgin olive oil with 400 mg/kg phenolic compounds (0.1 μmol/L) [22]. We tested the in vitro bioactivity of all Ps at this concentration, and at concentrations 50% lower (0.05 μmol/L), 250% higher (0.25 μmol/L), and 500% higher (0.5 μmol/L). Using as a reference a similar in vitro study with hydroxytyrosol [23], we also performed the same set of experiments with nutraceutical-like concentrations (1, 2, 5, and 10 μmol/L).

Concerning the Ts, we considered the maximal plasma concentration of oleanolic acid (the most studied triterpenic acid from olive oil) after a standard dietary intake of a virgin olive oil with 31.9 mg/kg of oleanolic acid. The concentration of 0.002 μmol/L was selected as the physiological reference [24]. In a similar strategy to that performed for Ps, we tested the in vitro bioactivity of the three Ts at the previous concentration (0.002 μmol/L) and at concentrations 50% lower (0.001 μmol/L), 250% higher (0.005 μmol/L), and 500% higher (0.01 μmol/L). We also tested their biological capacities at nutraceutical concentrations (0.25, 1, 2, and 5 μmol/L).

### 2.2. In Vitro Bioactivities

#### 2.2.1. Antioxidant Properties

We tested the extract capacity to counteract the oxidation of low-density lipoproteins (LDL) in a pro-oxidant environment (in the presence of a pro-oxidant agent, Cu^2+^) [25,26]. In brief, we obtained LDL from a pool of plasma from 20 healthy volunteers by sequential density gradient ultracentrifugation [27] and dialyzed it against 1× phosphate-buffered saline (Sigma-Aldrich, Barcelona, Spain). We mixed LDLs (final concentration: 10 mg/dL LDL cholesterol) with CuSO_4_ (final concentration: 5 μmol/L) in the presence of the Ps and Ts as the previously described. These combinations were incubated at 37 °C in 96-well transparent plates in an Infinite M200 reader (Tecan Ltd., Männedorf, Switzerland) for 4 h. We measured the absorbance at 234 nm (proportional to the levels of conjugated dienes in the samples, an indicator of LDL oxidation) every 3 min to obtain the kinetic curve of LDL oxidation in each condition. From the curves, we calculated: (1) the lag time (the time when the oxidation at maximal velocity started, in minutes) and (2) the oxidation rate (the speed of LDL maximal oxidation, the slope of the oxidation curve in the propagation phase as Δ absorbance/min). We performed experiments in triplicate and calculated the mean values for each extract. We calculated the change in both properties as its percentage difference relative to the negative control: (value for each extract—value for the negative control)/value for the negative control × 100.

#### 2.2.2. Vasoactive Properties

We tested the capacity of bioactive compounds to induce the release of nitric oxide from endothelial cells [28]. We cultured a line of human umbilical vein endothelial cells in supplemented EGM-2 medium (Lonza, Basel, Switzerland), refreshing them every 48–72 h. We detached cells using 0.025% trypsin for five minutes and seeded in 96-well plates to 80–90% confluence 24 h prior to the experiments. We then washed the cells, incubated them in fresh EGM-2 medium (now supplemented with 0.75% bovine serum albumin, 1% fetal calf serum, and 1% penicillin-streptomycin) with 5 μmol/L 4,5-diaminofluorescein (Sigma-Aldrich) and the nine extracts in the previously described concentrations (or without them, in the negative control of the experiment) at 37 °C for 1 h. The detection of nitric oxide is based on its capacity to react with 4,5-diaminofluorescein and produce a fluorescent signal. We measured fluorescence (Excitation/Emission: 485/532 nm) every 15 min in an Infinite M200 reader (Tecan Ltd., Männedorf, Switzerland), and computed the slope of the time-dependent increment in the fluorescent signal. We performed experiments in triplicate, calculated the mean value for each extract, and computed the change in the slope relative to the negative control as described for antioxidant properties.

We also assessed the capacity of extracts to inhibit the release of endothelin-1, a vasoconstrictor molecule. We incubated human umbilical vein endothelial cells seeded in 96-well plates with EGM-2 medium (supplemented with 0.75% bovin serum albumin, 1% fetal calf serum, and 1% penicillin-streptomycin) with 5 μmol/L tumor necrosis factor alpha as pro-inflammatory stimuli and the nine extracts (or without them, in the negative control of the experiment). Twenty-four hours later, we collected the supernatants and measured the concentration of endothelin-1 using an ELISA kit (Endothelin-1 ET-1 Human ELISA Kit, Thermo Fisher Scientific, Barcelona, Spain). We performed cell experiments in triplicate, combined the three supernatants, and quantified endothelin-1 in a single experiment for each extract. We calculated the change in endothelin-1 levels relative to the negative control as previously described for other biological activities.

#### 2.2.3. Anti-Inflammatory and Antithrombotic Properties

We measured anti-inflammatory and antithrombotic capacities of each extract as their capacity to decrease the in vitro endothelial release of vascular cell adhesion molecule-1 (VCAM-1) and plasminogen activator inhibitor-1 (PAI-1), respectively. To this purpose, we measured VCAM-1 and PAI-1 levels released by the lines of human umbilical vein endothelial cells after 24 h stimulation with 5 μmol/L tumor necrosis factor alpha as described in the endothelin-1 experiments. We quantified both using ELISA kits (VCAM-1 Human ELISA Kit, PAI-1 Human ELISA Kit; both from Thermo Fisher Scientific, Barcelona, Spain), and calculated their change relative to the negative control as previously described for other cell-based properties.

### 2.3. Evidence-Based Combination of Extracts

From the experiments at different doses, we first calculated the dose-dependent change in the in vitro properties (oxidation rate data were previously log-transformed). Due to their potentially beneficial nature, we positively scored increments in lag time values, decreases in oxidation rate (where data were previously log-transformed) [29], increases in nitric oxide release [30], and decrements in endothelin-1 [31], VCAM-1 [32], and PAI-1 values [33]. To calculate the scores, we sorted the nine extracts from highest to lowest protective effect and assigned nine points to the one with the greatest benefit, eight points to the second most beneficial, seven to the next, and so on until we reached the one with the least benefit. Extracts not presenting a beneficial dose-dependent response obtained zero points. Regarding the antioxidant score, given that lag time is its most representative parameter [29], this property was weighted twice than that of the improvement in oxidation rate. In relation to vasoactive capacities, enhancement in nitric oxide release was weighted twice than that of changes in endothelin-1 values. The sum score calculation was as follows: [(lag time score × 2) + oxidation rate]/3 + [(nitric oxide score × 2) + endothelin-1 score]/3 + VCAM-1 score + PAI-1 score.

The top five extracts according to sum score values were considered for combination. Mixtures were performed in two dose ranges: (1) “nutritional range” (around those achievable through dietary intake of a virgin olive oil, ~0.1 μmol/L); and (2) “nutraceutical range” (around the median of non-dietary doses, ~5 μmol/L). We tested the capacity of the combinations to improve in vitro bioactive properties, in a single experiment for each combination. To obtain concentration-corrected results (some of the combinations presented greater levels of bioactive compounds), we divided the changes in the properties by the total concentration of active components in the mix. Finally, to decide which combination presented the best in vitro profile, we sorted the four combinations from highest to lowest protective effect and assigned four points to the one with the greatest benefit, three points to the second most beneficial, and so on until we reached the one with the least benefit. Combinations not presenting a beneficial response obtained zero points.

## 3. Results

### 3.1. Dose-Dependent Bioactivities of Individual Extracts

As shown in Table 1, we only observed in vitro bioactivity in the case of Ps. We could sort Ps from highest to lowest antioxidant capacity as follows: P4 > P2 > P1 > P3 > P6 > P5. Regarding vasoactive ability, the sequence was: P2 > P5 > P1 > P4 > P3. In terms of anti-inflammatory capacity: P5 > P6 > P4 > P3 > P2 > P1. Finally, in relation to antithrombotic ability: P5 > P4. 

### 3.2. Combinations of Extracts

As has been previously described, the top five extracts according to their sum scores were considered for combinations. P5 (75% oleocanthal) was the best one in relation to anti-inflammatory and antithrombotic abilities, P4 (70% oleuropein) in relation to antioxidant capacity, and P2 (76% 3,4-dihydroxyphenylglycol) in relation to vasoactive potential. P1 was associated with a moderate antioxidant, vasoactive, and anti-inflammatory potential, and was also considered. Finally, P3 contained the most studied olive oil phenolic compound (95% hydroxytyrosol). It was prioritized over P6 (also containing hydroxytyrosol) because it presented a greater sum score and could be produced in larger quantities for use in industrial processes. Moreover, in contrast to P5, which was isolated by a laborious procedure not applicable to industrial purpose, P1, P2, P3, and P4 were easily obtained from olive mill waste.

Thus, combination 1 (C1) consisted of an isomolar mixture of P2, P3, and P4. Combination 2 (C2) consisted of an isomolar mixture of P2, P3, P4, and P5. Combination 3 (C3) consisted of an isomolar mixture of P1, P2, P3, and P4. Finally, combination 4 (C4) consisted of an isomolar mixture of P1, P2, P3, P4, and P5. The four combinations and their concentrations are shown in Table 2.

### 3.3. Effects of Extract Combinations

The combination with the best dose-adjusted in-vitro properties was #1. It scored the highest for antioxidant, vasoactive, and antithrombotic properties at both dose ranges, and for anti-inflammatory capacity at nutraceutical ones (Table 3).

**Scheme 1:**
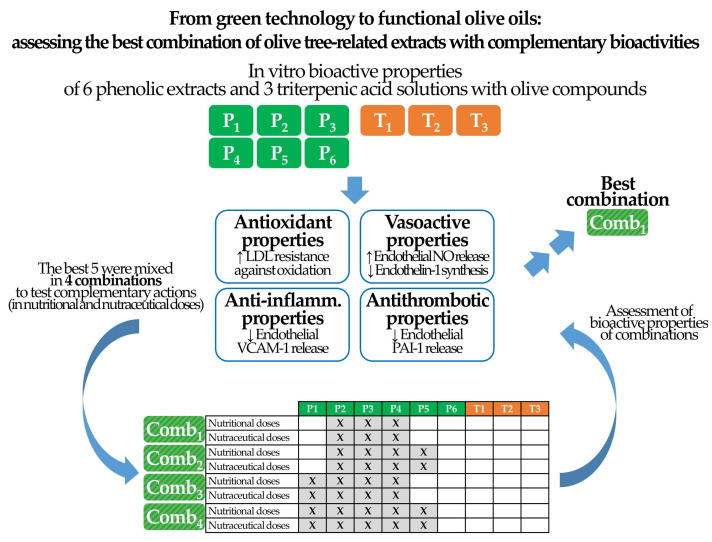
Approach for the selection of the best extract combination with complementary bioactivities.

Scheme 1 represents a summary of the strategy of our work and its main conclusion.

## 4. Discussion

The antioxidant, anti-inflammatory, anti-coagulant, and vasoactive bioactivities of several phenolic extracts from olive-tree related products were examined ex vivo. Selected combinations of extracts were re-tested for the aforementioned bioactivities in order to account for antagonisms and synergisms among them. Two sum scores, at dietary and nutraceutical doses, were applied with quite similar results. The final results point at combination 1, rich in 3,4-dihydroxyphenylglycol, hydroxytyrosol, and oleuropein, as the optimum in order to prepare functional products and nutraceuticals with cardioprotective properties. This is despite the fact that the isolated extract with greatest properties was P5 (rich in oleocanthal), suggesting a potential synergistic effect among different olive components.

There is great interest in the food industry in the development of functional foods, particularly highlighted in products such as olive oils with additional health properties [34,35,36,37]. The development of such foods, enriched in various bioactive components, can be optimized if based on scientific evidence to the extent possible, since the combination of different doses of these nutrients could lead to improved health effects. Regarding extract combinations with higher bioactivities in our data, the combination of extracts rich in 3,4-dihydroxyphenylglycol (P2), hydroxytyrosol (P3), and oleuropein (P4) resulted in the greatest improvement of the cardioprotective bioactivities in most of the experiments (antioxidant, vasoprotective, and anti-inflammatory properties). 3,4-dihydroxyphenylglycol [38,39], hydroxytyrosol [40,41], and oleuropein [42] have previously shown diverse protective effects in molecular mechanisms related to cardiovascular and other non-communicable disease, and the potential synergistic effect among them may play a role in the most complete protective action. These results agree with those shown by De Roos et al., in which a synergistic effect between hydroxytyrosol and 3,4-dihydroxyphenylglycol was observed in the inhibition of platelet aggregation [43]. Combination #2 (combination #1 plus P5) a priori should have been the most complete bioactive combination since it provides the compounds with greater antioxidant and vasoprotective effects (P2, P3, P4), and the oleocanthal-rich extract, the one with greater anti-inflammatory and anticoagulant effects in our data. Oleocanthal has been attributed several protective capacities on health outcomes in previous bibliography [44]. However, this mixture does not present additive effects in many of the properties, only scores higher than combination #1 on the mixture’s ability to decrease the in vitro endothelial secretion of endothelin-1 and VCAM-1, and presents the additional limitation that its production at industrial scale is not currently feasible. Finally, regarding combinations #3 and #4 (combination #1 plus P1, and combination #1 plus P1 and P5, respectively), they were designed to capture the contribution of the possible synergic effects of P1. It was composed of modest amounts of different compounds, many of its components were present in higher amounts in other tested extracts (3,4-dihydroxyphenylglycol in P2, hydroxytyrosol in P3, and oleuropein in P4), and it was initially considered under the hypothesis that the biological effects observed could be produced by synergy among compounds rather than by a specific molecule. However, when compared to the other combinations, their effects per mole of phenolic compounds were lower. As observed in previous studies on phenolic compounds [45,46], these results highlight the relevance of testing the combination of active principles when preparing a functional food or nutraceutical.

Triterpenic acid effects were also tested in vitro due to their presence in virgin olive oil although in modest amounts and their association with an improvement of endothelial function in vitro and in humans [47]. However, triterpenic acids showed no remarkable in vitro biological properties compared to the other extracts. The lack of direct antioxidant effects on LDLs could be partially explained because these compounds are not able to donate electrons directly as phenolic compounds do [48]. In relation to the rest of in vitro properties, the in vivo transformation of triterpenes into secondary metabolites able to protect endothelial cells cannot be discarded. In addition, bioactive molecules, when outside their natural food matrix, might not be properly incorporated to the media and therefore may have not accessed the cells [49].

The consumption of a natural virgin olive oil of around 400 mg/kg promotes a maximum plasma concentration of 0.1 μmol/L of hydroxytyrosol [22]. Thus, we hypothesize that the consumption of a functional olive oil enriched with olive bioactive substances (acceptable palatability: 1000 mg/kg [50]) would produce a plasma concentration of phenolic compounds around 0.25 μmol/L. Nevertheless, biological effects in the in vitro experiments were sometimes observed at concentrations considerably higher than real-life natural virgin olive oil consumption. The lowest concentration with bioactivity in some of the tests was 5 μmol/L. Therefore, we designed extract combinations at two concentration ranges, functional and nutraceutical, in order to overcome this limitation and to be able to capitalize on the potential biological effects that could be derived from the combinations of compounds that we tested. In agreement with our working hypothesis, our findings resulted in a phenolic extract combination that could be employed for the elaboration of a functional olive oil, which was successfully tested in animal experiments. In this regard, a chronic sustained treatment with an extra-virgin olive oil enriched with bioactive compounds from the olive fruit and leaves (750 mg/kg of phenolic compounds, mainly hydroxytyrosol, 3,4-dihydroxyphenylglycol, and oleuropein) was an effective strategy for reducing blood pressure and circulating cholesterol in spontaneously hypertensive rats [51]. These results validate our strategy for selecting the best combination of extracts to prepare functional olive oils. In addition, they encourage the development of functional virgin olive oils to improve specific health effects.

This work has strengths and limitations. The study presents an evidence-based design of a palatable and functional olive oil from olive fruit and leaf-related extracts with enhanced in vitro bioactivities. Sum scores permitted the selection of a complementary combination of olive oil minor components, which may have industrial-scale applications. Finally, these extracts can be obtained from olive tree waste products, which would contribute to minimizing industrial waste, save energy, and be environmentally sustainable. However, our study also has limitations. First, although equivalent concentrations were established, the pre-analytic experimental conditions could have played a role in the differences observed among extracts. Second, enzyme immune-assays were only performed in single experiments so that different extract concentrations could be measured in the same analytical run and increase comparability. However, the in vitro and cellular experiments were performed in triplicate. Third, two extracts (P5 and P6) presented some undetermined compound in their chemical profile (as indicated by their spectra), thus other biochemical mechanisms for the observed effects cannot be discarded. Finally, the use of cellular models may not determine the contra-regulatory mechanisms present in vivo.

## 5. Conclusions

The extract combination with the best overall improvement of the bioactivities examined (antioxidant, vasoactive, anti-inflammatory, and anticoagulant), at functional doses, was the combination of 3 different extracts, rich in 3,4-dihydroxyphenylglycol, hydroxytyrosol, and oleuropein. Based on the in vitro effects, the combination of functional extracts at 0.15 μmol/L (600 mg/kg equivalents of hydroxytyrosol if we consider 0.05 μmol/L, 200 mg/kg, of each extract) would be appropriate to elaborate a palatable and functional olive oil. The extract containing oleocanthal (P5) requires further study, as it was individually associated with the greatest improvements in the in vitro properties (nevertheless, research into an oleocanthal extraction applicable at an industrial level is required for its consideration in future functional oils). Our work suggests a potential strategy for the evidence-based design of a functional food.

## 6. Patents

Phenolic extract #2 (P2) was obtained from olive leaves using the method described in the patent PCT/ES2012/070491 [16]. Phenolic extract #3 (P3) was obtained from the liquid phase of *alperujo* after solid/liquid separation by the patented system PCT/ES2002/00058 [17,18]. Procedures for the elaboration of phenolic extracts #1 (P1) and #4 (P4) are currently in the process of being patented. Phenolic extract #6 (P6) was obtained from olive oil waste water using the method described in the patent PCT/ES2012/070491 [16].

## Figures and Tables

**Table 1:** Dose-dependent associations of phenolic (0.05–10 μmol/L) and triterpenic solutions (0.001–10 μmol/L) with in vitro bioactive properties ^1^.

	Antioxidant Properties					Vasoactive Properties					Anti-Inflammatory Properties		Anticoagulant Properties		Score Sum
	Increase in LDL Lag Time		Decrease in LDL Oxidation Rate		Weighted Score	Increase in Nitric Oxide Release		Decrease in Endothelin-1 Release		Weighted Score	Decrease in VCAM-1 Release		Decrease in PAI-1 Release		
	% [95% CI]	Score	% [95% CI]	Score		% [95% CI]	Score	% [95% CI]	Score		% [95% CI]	Score	% [95% CI]	Score	
P1	168	7	−67.4	8	7.33	10.3	9	−0.33	0	6	−0.97	4	−1.05	0	17.33
	[133; 203]		[−79.1; −55.7]			[8.79; 11.8]		[−2.63; 1.97]			[−1.15; −0.78]		[−2.94; 0.85]		(4th)
P2	171	8	−59.7	7	7.67	9.77	8	−0.94	8	8	−1.62	5	−0.47	0	20.67
	[145; 197]		[−66.5; −52.9]			[8.71; 10.8]		[−1.27; −0.62]			[−2.19; −1.05]		[−1.37; 0.43]		(3rd)
P3	70.8	6	−26.9	6	6	−0.2	0	−0.39	7	2.33	−1.93	6	0.41	0	14.33
	[34.8; 107]		[−43.2; −10.6]			[−0.74; 0.33]		[−0.65; −0.12]			[−3.27; −0.60]		[−0.43; 1.25]		(5th)
P4	210	9	−71.1	9	9	4.75	7	0.33	0	4.67	−2.05	7	−0.51	8	28.67
	[126; 294]		[−92.7; −49.5]			[4.11; 5.40]		[−0.49; 1.16]			[−2.45; −1.66]		[−0.90; −0.11]		(2nd)
P5	21.9	4	−12.2	5	4.33	0.86	6	−4.8	9	7	−3.74	9	−5.78	9	29.33
	[19.7; 24.1]		[−17.4; −6.91]			[0.51; 1.21]		[−6.17; −3.43]			[−7.22; −0.26]		[−6.29; −5.27]		(1st)
P6	67.3	5	−11.1	4	4.67	−0.39	0	0.024	0	0	−2.49	8	1.51	0	12.67
	[35.5; 99.0]		[−12.6; −9.61]			[−0.88; 0.087]		[−0.32; 0.37]			[−4.71; −0.28]		[0.52; 2.50]		(6th)
T1	−0.62	0	1.17	0	0	0.24	0	0.77	0	0	0.96	0	−0.76	0	0
	[−1.07; −0.17]		[0.40; 1.94]			[−0.36; 0.84]		[0.40; 1.14]			[−2.38; 4.30]		[−4.94; 3.42]		(7th)
T2	−0.042	0	0.82	0	0	−0.22	0	−0.96	0	0	−1.51	0	−0.66	0	0
	[−0.33; 0.25]		[0.31; 1.32]			[−0.52; 0.070]		[−3.12; 1.20]			[−4.62; 1.60]		[−1.47; 0.15]		(7th)
T3	−0.042	0	1.13	0	0	−0.13	0	0.79	0	0	−2.27	0	−2.59	0	0
	[−0.33; 0.25]		[−0.030; 2.30]			[−0.97; 0.71]		[0.59; 0.99]			[−8.95; 4.40]		[−7.22; 2.04]		(7th)

^1^*PAI-1*: plasminogen activator inhibitor-1; *P*: phenolic extract; *T*: triterpenic acid solution; *VCAM-1*: vascular cell adhesion molecule-1.

**Table 2:** Extract combination strategy ^1^.

		P1 (μmol/L)	P2 (μmol/L)	P3 (μmol/L)	P4 (μmol/L)	P5 (μmol/L)	P6 (μmol/L)	T1 (μmol/L)	T2 (μmol/L)	T3 (μmol/L)	Sum
C1	Nutritional doses		0.05	0.05	0.05						0.15 μmol/L
	Nutraceutical doses		1	1	1						3 μmol/L
C2	Nutritional doses		0.05	0.05	0.05	0.05					0.2 μmol/L
	Nutraceutical doses		1	1	1	1					4 μmol/l
C3	Nutritional doses	0.05	0.05	0.05	0.05						0.2 μmol/L
	Nutraceutical doses	1	1	1	1						4 μmol/l
C4	Nutritional doses	0.05	0.05	0.05	0.05	0.05					0.25 μmol/L
	Nutraceutical doses	1	1	1	1	1					5 μmol/L

^1^*C*: combination; *P*: phenolic extract; *T*: triterpenic acid solution.

**Table 3:** Effects of extract combinations ^1^.

	Antioxidant properties					Vasoactive properties					Anti-Inflammatory Properties		Anticoagulant Properties		Score Sum
	Increase in LDL Lag Time		Decrease in LDL Oxidation Rate		Weighted Score	Increase in Nitric Oxide Release		Decrease in endothelin-1 Release		Weighted Score	Decrease in VCAM-1 Release		Decrease in PAI-1 Release		
	%	Score	%	Score		%	Score	%	Score		%	Score	%	Score	
Nutritional doses															
C1	606.1	4	−14.3	4	4	3.7	4	−6.0	2	3.33	2.5	0	−5.3	4	11.33 (1st)
C2	287.6	2	−12.4	2	2	1.8	1	−8.9	4	2.00	−14.4	4	−5.0	3	11.00 (2nd)
C3	390.4	3	−12.5	3	3	2.2	3	−2.7	1	2.33	0.9	0	5.8	0	5.33 (4th)
C4	158.6	1	−12.0	1	1	2.1	2	−8.4	3	2.33	−8.9	3	−3.6	2	8.33 (3rd)
Nutraceutical doses															
C1	180.6	1	−48.2	4	2.00	40.3	4	−35	3	3.67	−53.8	4	−77.3	4	13.67 (1st)
C2	181.9	2	−38.7	2.5	2.17	24.4	3	−7.6	2	2.67	−32.9	3	−24.8	3	10.83 (2nd)
C3	237.8	4	8.7	0	2.67	15.6	2	18.9	0	1.33	155.9	0	728.8	0	4.00 (4th)
C4	193.8	3	−38.7	2.5	2.83	7.2	1	−53	4	2.00	37.8	0	−6.6	2	6.83 (3rd)

^1^*C*: combination; *PAI-1*: plasminogen activator inhibitor-1; *PE*: phenolic extract; *VCAM-1*: vascular cell adhesion molecule-1. C1: P2 + P3 + P4; C2: P2 + P3 + P4 + P5; C3: P1 + P2 + P3 + P4; C4: P1 + P2 + P3 + P4 + P5.

## Data Availability

The data presented in this study are fully available in the Results.
